# Frailty Prevalence and Characterization Among Kidney Transplant Candidates in Spain: A Multicenter Study

**DOI:** 10.3389/ti.2025.14098

**Published:** 2025-06-24

**Authors:** María José Pérez-Sáez, Edoardo Melilli, Marta Arias, Antonio Franco, Rocío Martínez, Asunción Sancho, María Molina, Carme Facundo, Natalia Polanco, Verónica López, Auxiliadora Mazuecos, Sheila Cabello, María Elena González García, María Luisa Suárez, Ingrid Auyanet, Jordi Espi, Teresa García Falcón, Juan Carlos Ruiz, Cristina Galeano, Marta Artamendi, María Luisa Rodríguez-Ferrero, José María Portolés, María Auxiliadora Santana, Paloma Leticia Martín-Moreno, Nisrine Arhda, Marta Calvo, Alicia Mendiluce, Manuel Macía, María Lourdes Pérez-Tamajón, Javier de Teresa, Blanca Gascó, Sagrario Soriano, Guadalupe Tabernero, Lourdes de la Vara, Ana María Ramos, Rafael Martínez, Enrique Montero de Espinosa, José Luis Zalve, Julio Pascual, Leocadio Rodríguez-Mañas, Alex Gutiérrez-Dalmau, Francesc Moreso

**Affiliations:** ^1^ Nephrology Department, Hospital del Mar, RICORS2040 RD21/0005/0022, Barcelona, Spain; ^2^ Hospital del Mar Research Institute, Barcelona, Spain; ^3^ Nephrology Department, Hospital Universitari de Bellvitge, L’Hospitalet de Llobregat, Barcelona, Spain; ^4^ Nephrology Department, Hospital Clínic, Barcelona, Spain; ^5^ Nephrology Department, Hospital General Universitario, Alicante, Spain; ^6^ Nephrology Department, Hospital Universitario Infanta Cristina, Badajoz, Spain; ^7^ Nephrology Department, Hospital Universitario Doctor Peset, Valencia, Spain; ^8^ Nephrology Department, Hospital Universitari Germans Trias i Pujol, RICORS2040, Barcelona, Spain; ^9^ Nephrology Department, Fundació Puigvert, Institut de Recerca Sant Pau (IR Sant Pau), RICORS2040, Barcelona, Spain; ^10^ Nephrology Department, Hospital 12 de Octubre, Madrid, Spain; ^11^ Nephrology Department, Hospital Regional Universitario de Málaga, Málaga, Spain; ^12^ National Network for Kidney Research RICORS2040 RD21/0005/0012, Instituto Biomédico de Investigación de Málaga (IBIMA), Universidad de Málaga, Málaga, Spain; ^13^ Nephrology Department, Hospital Universitario Puerta del Mar, Cádiz, Spain; ^14^ Nephrology Department, Hospital Universitari Son Espases, Palma de Mallorca, Spain; ^15^ Nephrology Department, Hospital Universitario La Paz, Madrid, Spain; ^16^ Nephrology Department, Hospital Universitario Central de Asturias, Oviedo, Spain; ^17^ Nephrology Department, Complejo Hospitalario Insular de Gran Canaria, Las Palmas de Gran Canaria, Spain; ^18^ Nephrology Department, Hospital Universitario y Politécnico La Fe, Valencia, Spain; ^19^ Nephrology Department, Complejo Hospitalario Universitario A Coruña, A Coruña, Spain; ^20^ Nephrology Department, Hospital Universitario Marqués de Valdecilla, Instituto de Investigación Valdecilla (Idival), Santander, Spain; ^21^ Nephrology Department, Hospital Universitario Ramón y Cajal, Instituto Ramón y Cajal de Investigación Sanitaria (IRyCIS), Madrid, Spain; ^22^ Nephrology Department, Hospital San Pedro, La Rioja, Spain; ^23^ Nephrology Department, Hospital General Universitario Gregorio Marañón, Madrid, Spain; ^24^ Nephrology Department, Hospital Universitario Puerta de Hierro, Madrid, Spain; ^25^ Nephrology Department, Hospital Universitario de Cruces, Barakaldo, Bizkaia, Spain; ^26^ Nephrology Department, Clínica Universidad de Navarra, Navarra Institute for Health Research (IdiSNA), Pamplona, Spain; ^27^ Nephrology Department, Hospital Clínico Universitario de Santiago, Santiago de Compostela, Spain; ^28^ Nephrology Department, Hospital Clínico San Carlos, Madrid, Spain; ^29^ Nephrology Department, Hospital Clínico Universitario de Valladolid, Valladolid, Spain; ^30^ Nephrology Department, Hospital Virgen de la Candelaria, Santa Cruz de Tenerife, Spain; ^31^ Nephrology Department, Hospital Universitario de Canarias, Santa Cruz de Tenerife, Spain; ^32^ Nephrology Department, Hospital Universitario Virgen de las Nieves, Granada, Spain; ^33^ Nephrology Department, Hospital Virgen del Rocío, Sevilla, Spain; ^34^ Nephrology Department, Hospital Universitario Reina Sofía, Córdoba, Spain; ^35^ Nephrology Department, Hospital Universitario de Salamanca, Instituto de Investigación Biomédica de Salamanca (IBSAL), Salamanca, Spain; ^36^ Nephrology Department, Complejo Hospitalario General Universitario de Albacete, Albacete, Spain; ^37^ Nephrology Department, Fundación Jiménez Díaz, Madrid, Spain; ^38^ Sandoz Farmacéutica SA, Head of Medical Department, Madrid, Spain; ^39^ Geriatrics Department, Hospital Universitario de Getafe, Madrid, Spain; ^40^ Nephrology Department, Hospital Universitario Miguel Servet, Zaragoza, Spain; ^41^ Nephrology Department, Hospital Universitario General Vall d’ Hebron, Barcelona, Spain

**Keywords:** FRAIL, frailty, kidney transplant, waiting list, candidate

## Abstract

Frailty is a frequent condition among kidney transplant candidates (KTc) that confers poor outcomes after transplantation. We aimed to establish frailty prevalence in a representative sample of KTc in Spain. We conducted a multicenter cross-sectional study including 1194 KTc ≥50 years. Frailty was assessed by the FRAIL scale. Mean age was 64.2 years; 38.4% were female. Median Charlson comorbidity index (CCI) was 6 [4–7] and the total number of medications was 9 [7–12]. We found that 8.2% of patients were frail and 41.5% were pre-frail. Frailty was more frequent among females (60.2% of frail vs. 32.8% of robust; p < 0.001), hemodialysis patients (74.5% of frail vs. 67.1% of robust; p = 0.02), and those with a high burden of disease (54.6% of frail patients with CCI >6 vs. 29.3% of robust; p < 0.001). The multivariable analysis confirmed that frailty was associated with the female sex (OR 3.9 [2.5–6.2]); higher CCI (>6 OR 2.9 [1.6–54]); and the number of medications (OR –per medication- 1.13 [1.07–1.2]). Almost 50% of KTc in Spain are pre-frail or frail. Frailty is more prevalent between women and patients with high comorbidity burden. Identifying those candidates at risk is essential to establish risks and implement strategies to minimize them.

## Graphical Abstract

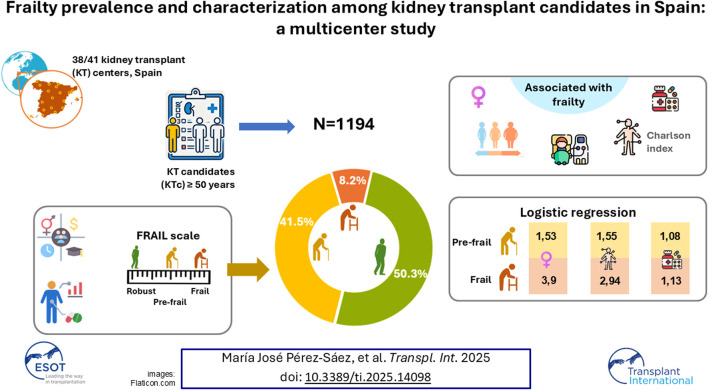

## Introduction

Frailty is characterized by a reduced physiological reserve to stressors and was initially studied within the aging population residing in communities [1]. Among individuals with advanced chronic kidney disease (CKD), frailty is a frequent condition and has been reported to affect up to 70% of patients receiving hemodialysis [2, 3]. These patients experience poorer outcomes while on dialysis, including higher mortality rates [4, 5].

Frail CKD patients have also restricted access to the kidney transplantation (KT) waiting list and their chances of receiving a transplant are notably reduced [6, 7]. Among subjects evaluated for KT, frailty prevalence ranges from 5% to more than 50%, depending on the series and the scale used [6–9]. Eventually, pooled analysis of different studies shows that about 17% of KT recipients are identified as frail [10]. However, most of the studies included in the systematic reviews of frailty among KT recipients come from US cohorts, with a very small representation of European studies [11]. Sociodemographic differences between American and European populations prevent direct extrapolation of the results.

Frail KT recipients experience heightened rates of complications such as intolerance to immunosuppressants [12], prolonged length of stay and a higher rate of readmissions [13, 14], higher rate of delayed graft function and surgical complications [15, 16], and, more importantly, higher post-transplant mortality [14, 17–21].

In Spain, fewer than 20% of dialysis patients have access to KT [22]. Possibly, frailty hampers this access, especially among elderly recipients. Despite the recognized impact of frailty on KT outcomes, clinicians often encounter challenges in assessing frailty during outpatient visits, and questions arise regarding the best scale to use and the potential utility of the information obtained [23]. A survey across 133 KT programs in the US revealed that 69% of centers reported performing standardized frailty assessments during transplant evaluations, yet there was little consensus on the preferred tool for measuring frailty [24]. The scale proposed by Linda Fried more than 20 years ago, the Physical Frailty Phenotype (PFP), has emerged as the most used frailty scale in research involving KT candidates and recipients [1]. However, other less time-consuming metrics, like the FRAIL scale, have also found utility in this context [7, 25]. Centers conducting frailty evaluations through validated tools have demonstrated better waitlist and transplant outcomes, regardless of the tool used [26]. Although correlation among different frailty metrics is poor [27–29], identifying patients at risk for unfavorable results holds paramount importance in assessing prognosis, establishing preventive strategies, and implementing therapeutic interventions such as prehabilitation.

This is a multicenter cross-sectional study carried out with the participation of the vast majority of KT Units in Spain. We aimed to establish frailty prevalence and associated factors among KT candidates over 50 years in our setting, as well as to boost the universal implementation of the frailty measurement as part of the KT candidacy study work-up.

## Patients and Methods

### Study Design and Participants

This is a multicenter, cross-sectional study carried out in 38 KT Units in Spain during 2022.

All KT Units in Spain were invited to participate and 38 out of 41 agreed. During the outpatient visits, subjects ≥50 years old included on the KT waiting list and able to consent were invited to participate in the study. Both patients already included on the waiting list and those who were new inclusions during the visit could be included in the study. Patients with a major psychiatric disorder, cognitive impairment, or an acute condition that to the judgment of the investigator could cause a physical impairment were excluded from the study.

The study started in March 2022 and the inclusion was competitive among centers until the end of the study (December 2022). The number of patients included was different across centers, depending on the number of patients included on their KT waiting list, the frequency of the visits, etc. Although there were differences, with a maximum of 169 and a minimum of 2 patients per center, 50% centers included more than 20 patients in the study.

Clinical and epidemiological variables and the FRAIL scale were collected at each center and introduced in a central database. Data extraction and analysis were further conducted.

### Ethics

The Institutional Review Board of Hospital del Mar approved the study (2020/9349), and all enrolled participants provided written informed consent at the time of frailty evaluation. The study followed the principles of the Declaration of Helsinki, only relying on the official database.

### Frailty Assessment

Frailty was assessed according to the FRAIL scale which includes 5 questions (all of them self-reported) assessing fatigue, resistance, ambulation, illness, and loss of weight. In both scales, each component or question scores 0/1 depending on its presence or absence. Robust patients were defined by a score of 0, pre-frail as those who ranked 1–2, and frail patients were defined by a score ≥3 [30].

The FRAIL scale has been proposed as a screening tool for frailty in general population [31]. It has been used in Spanish geriatric population [32] but also in Spanish KT candidates [7, 28].

### Study Variables

Besides the FRAIL scale, we included demographics (age, sex, ethnicity); social (education -defined by 4 categories: elementary, primary education, secondary education, and tertiary education-, family or social support –living by their own, in family, with friends, in a health/social facility-); and clinical data (body mass index (BMI), Charlson comorbidity index (CCI) [33], total number of medications, cause of renal disease, type of renal replacement therapy (RRT), date of dialysis initiation and date of waiting list inclusion, candidate to re-transplantation, albumin levels, C-reactive protein levels).

### Statistics

Continuous variables were expressed as mean ± standard deviation (SD), or median and interquartile range (IQR), according to normal distribution. Categorical data were expressed as absolute numbers and percentages. Comparisons of baseline characteristics between two groups were made using Chi-square or Fisher’s exact tests to analyze categorical variables, Student’s t-test for continuous variables with normal distribution, and Mann–Whitney test for non-parametric variables. When three categories were present, the Chi-square test was also used to compare categorical variables, the ANOVA test to compare quantitative variables with normal distribution, and the Kruskal-Wallis test for quantitative variables without normal distribution. Binomial and multinomial logistic regression models considering frailty as yes/no (merging pre-frailty and frailty status) or with the three categories (robust, pre-frail, and frail) were conducted. Variables were considered to be included in the multinomial model if a p-value ≤0.20 was found in the bivariate analysis. Two multinominal logistic regression models were conducted: one including the global CCI (ranking 0–24), and other including only cardiovascular disease as Charlson comorbidities (ranking 0 to 4: myocardial infarction, congestive heart disease, peripheral vascular disease and cerebrovascular disease). Statistical analysis was performed using SPSS version 29 software (IBM, Armonk, NY, USA). P-values <0.05 were considered statistically significant.

## Results

A total number of 1194 KT candidates ≥50 years old were included in the study. Table 1 displays the main characteristics of the cohort. The mean age was 64.2 years, 38.4% of them were female and 92.7% were Caucasian. In terms of education and social support, 17.4% declared themselves as having received elementary education and 11.7% lived on their own. The most frequent cause of CKD was unknown (23.9%) followed by glomerular disease (19.5%). Almost one-third of candidates had received at least one kidney transplant before (27.6%). In terms of RRT modality, 64.9% were on hemodialysis, 18% were on peritoneal dialysis and 19.8% were on a situation of advanced CKD pre-dialysis. The median time from dialysis onset to the waiting list entry was 12 months. KT candidates presented with a high comorbidity burden (CCI >6) in 35.6% of the cases. Subsequently, the total number of medications prescribed to each patient was high (9). In terms of laboratory parameters, mean albumin levels were 4 g/dL and mean levels of C-reactive protein were 0.6 mg/dL.

**TABLE 1:** Baseline and clinical characteristics of the 1194 KT candidates included in the study.

	KT candidates (n = 1,194)
Age (years, mean ± sd) *n = 0, 0%	64.2 ± 8.4
Sex (female, n (%)) *n = 0, 0%	459 (38.4)
Ethnicity (Caucasian, n (%)) *n = 9, 0.8%	1,107 (92.7)
Education (elementary, n (%)) *n = 170, 14.2%	208 (17.4)
Family/social support (living alone, (%)) *n = 84, 7%	140 (11.7)
BMI (Kg/m^2^, mean ± sd) *n = 79, 6.6%	26.7 ± 4.5
Cause of renal disease *n = 5, 0.4% Unknown Diabetic nephropathy Glomerular disease Others	285 (23.9) 183 (15.3) 233 (19.5) 493 (41.3)
Previous KT (yes, n, (%)) *n = 14, 1.2% Number of previous KT (median [max-min])	329 (27.6) 1 [1–5]
Renal replacement therapy modality (n, (%)) *n = 3, 0.3% Hemodialysis Peritoneal dialysis Preemptive transplant	775 (64.9) 215 (18) 201 (19.8)
Time from dialysis onset to WL entry (years, median [IQR]) *n= 291, 24.7% Time from dialysis onset to frailty determination (years, median [IQR]) *n = 0, 0%	1 [0.5–1.9] 2 [1–3.9]
Charlson comorbidity index (median [IQR]) *n = 18, 1.5% Low comorbidity = 3–4 Intermediate comorbidity = 5–6 High comorbidity >6	6 [4–7] 337 (28.7) 420 (35.7) 419 (35.6)
Total number of different medications (median [IQR]) *n = 0, 0%	9 [7–12]
Albumin (g/dL, mean ± sd) *n = 200, 16.7%	4 ± 0.6
CRP (mg/dL, median [IQR]) *n = 370, 31%	0.6 [0.2–1.9]

KT, kidney transplant; sd, standard deviation; BMI, body mass index; IQR, interquartile range; WL, waiting list; CRP, C-reactive protein. *Frequencies and % of missing data of each variable.

**FIGURE 1:**
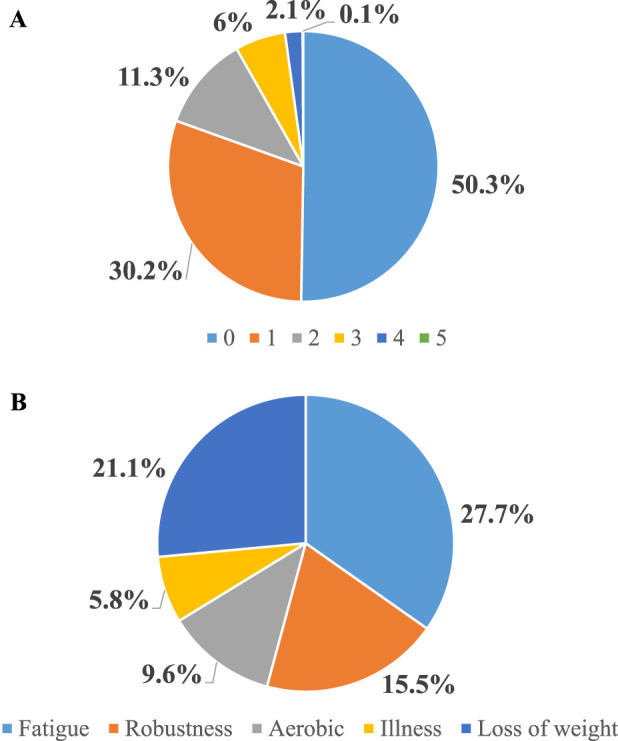
**(A)** Frailty prevalence according to the FRAIL scale among 1194 KT candidates in Spain. **(B)** FRAIL items distribution.

Frailty prevalence was determined by the FRAIL scale. Half of the patients were robust (50.3%), 41.5% were pre-frail, and 8.2% were frail. The most frequently reported item was fatigue (27.7%), followed by loss of weight (21.1%) and lack of robustness (15.5%). Figure 1.

Table 2 compares KT candidates who were robust, pre-frail, and frail. We found a higher percentage of females as the frail score increases (32.8% of robust patients; 40.9% of pre-frail; and 60.2% of frail patients). Frail candidates were also slightly more overweighted (BMI 27.5 kg/m^2^ in frail candidates vs. 26.1 kg/m^2^ in robust ones), were more frequently receiving hemodialysis as RRT (74.5% -frail- vs. 67.1% -robust-), had higher comorbidity burden (CCI >6 54.6% -frail- vs. 29.3% -robust-), and were on more medications (11 –frail- vs. 8.5 –robust-). On the contrary, the mean age was similar among robust and frail candidates. No differences in terms of albumin or C-reactive protein levels were found between robust or frail patients either.

**TABLE 2:** Baseline and clinical characteristics of KT candidates according to their FRAIL score.

Baseline and clinical characteristics of KT candidates	Robust group	Pre-frail group	Frail group	p-value
	FRAIL = 0 (n = 600)	FRAIL = 1–2 (n = 496)	FRAIL ≥3 (n = 98)	
Age (years, mean ± sd)	64.1 ± 8.2	64.5 ± 8.7	63.3 ± 8.1	0.475
Sex (female, n (%))	197 (32.8)	203 (40.9)	59 (60.2)	<0.001
Ethnicity (Caucasian, n (%))	553 (93.3)	464 (93.9)	90 (91.8)	0.729
Education (basic, n (%))	99 (19.4)	87 (20.3)	22 (26.2)	0.093
Family/social support (living alone, (%))	70 (12.6)	53 (11.4)	17 (18.5)	0.230
BMI (Kg/m^2^, mean ± sd)	26.1 ± 4.1	27.1 ± 4.6	27.5 ± 5.4	0.002
Cause of renal disease Unknown Diabetic nephropathy Glomerular disease Others	128 (21.5) 88 (14.8) 116 (19.5) 268 (44.6)	129 (26.1) 79 (16) 98 (19.8) 190 (19.8)	28 (28.6) 16 (16.3) 19 (19.4) 35 (35.7)	0.734
Previous KT (yes, n, (%)) Number of previous KT (median [max-min])	167 (28.1) 1 [1–3]	129 (26.4) 1 [1–5]	33 (34.4) 1 [1–3]	0.276 0.993
Renal replacement therapy modality (n, (%)) Hemodialysis Peritoneal dialysis Preemptive transplant	402 (67.1) 106 (17.7) 91 (15.2)	300 (60.7) 93 (18.8) 101 (20.4)	73 (74.5) 16 (16.3) 9 (9.2)	0.020
Time from dialysis onset to WL entry (years, median [IQR]) Time from dialysis onset to frailty determination (years, median [IQR])	1.1 [0.6–2.3] 2 [1–3.8]	1.2 [0.6–2.3] 1.8 [1–3.8]	1.5 [0.7–4.1] 3 [1.-4.9]	0.105 0.471
Charlson comorbidity index Low comorbidity = 3–4 Intermediate comorbidity = 5–6 High comorbidity >6	195 (33.2) 221 (37.6) 172 (29.3)	124 (25.3) 173 (35.2) 194 (39.5)	18 (18.6) 26 (26.8) 53 (54.6)	<0.001
Charlson comorbidity index (only considering CV risk factors (0–4) Low comorbidity = 0 Intermediate comorbidity = 1 High comorbidity =2–4	394 (55.9) 138 (42.6) 56 (9.52)	282 (40) 149 (46) 60 (12.2)	29 (4.1) 37 (11.4) 31 (31.9)	<0.001
Total number of different medications (median [IQR])	8.5 [7–11]	10 [7–12]	11 [8–13]	<0.001
Albumin (g/dL, mean ± sd)	4 ± 0.6	4 ± 0.5	3.9 ± 0.5	0.688
CRP (mg/dl, median [IQR])	0.6 [0.2–1.8]	0.5 [0.2–1.7]	1 [0.2–2.6]	0.205

KT, kidney transplant; sd, standard deviation; BMI, body mass index; IQR, interquartile range; WL, waiting list; CV, cardiovascular; CRP, C-reactive protein.

Two multinomial logistic regression analyses were conducted to analyze factors associated with pre-frailty and frailty in KT candidates (Table 3). In the first model, including global Charlson index as comorbidity index, female sex (odds ratio (OR) 1.53 [1.19–1.98]), high comorbidity burden (OR 1.55 [1.13–2.13]), and total number of medications (OR 1.08 per medication [1.04–1.12]) were associated with pre-frailty status. The same factors with higher intensity were also associated with frailty: female sex (OR 3.90 [2.46–6.19]), high comorbidity burden (OR 2.93 [1.61–5.41]), and total number of medications (OR 1.13 per medication [1.07–1.20]), Table 3. The second model included only cardiovascular disease (myocardial infarction, congestive heart disease, peripheral vascular disease and cerebrovascular disease) as comorbidity burden. Cardiovascular disease was highly associated with frailty in this cohort, starting at one cardiovascular problem (OR 3.46 [2.02–5.95], and increasing this association along with the number of cardiovascular problems (Table 3).

**TABLE 3:** Multinomial logistic regression of factors associated with pre-frailty and frailty in KT candidates. A) Considering global Charslon index; B) Considering a cardiovascular Charlson index (0–4).

Independent variables	OR	95% CI	
A			
Pre-frailty (FRAIL = 1–2)			
Sex (ref: male)	1.531	1.186	1.978
Charlson index (ref: 3–4)			
5–6	1.097	0.806	1.492
>6	1.550	1.126	2.135
Number of medications (per each one)	1.081	1.045	1.118
Frailty (FRAIL ≥3)			
Sex (ref: male)	3.904	2.462	6.190
Charlson index (ref: 3–4)			
5–6	1.030	0.539	1.970
>6	2.935	1.612	5.412
Number of medications (per each one)	1.132	1.067	1.200
B			
Pre-frailty (FRAIL = 1–2)			
Sex (ref: male)	1.504	1.166	1.941
Charlson CV index (ref: 0)			
1	1.387	1.043	1.844
2	1.369	0.876	2.139
3	1.369	0.541	3.462
4	1.416	0.196	10.247
Number of medications (per each one)	1.084	1.049	1.122
Frailty (FRAIL ≥3)			
Sex (ref: male)	4.117	2.572	6.591
Charlson CV index (ref: 0)			
1	3.466	2.019	5.948
2	8.204	4.278	15.731
3	7.897	2.337	26.682
4	7.897	2.337	26.682

KT, kidney transplant; CI, confidence interval; OR, odds ratio.

We initially explored factors associated with pre-frailty and frailty through a binomial logistic regression. Factors with a p-value <0.2 were included in the final multinomial analysis: A) age, sex, body mass index, level of education, renal replacement therapy modality, C-reactive protein levels, Charlson comorbidity index, and number of medications; B) age, sex, body mass index, level of education, renal replacement therapy modality, C-reactive protein levels, Charlson cardiovascular comorbidity index, and number of medications.

## Discussion

Herein, we present a multicenter cross-sectional study involving thirty-eight out of the total forty-one Kidney Transplant Units in Spain and more than 1000 CKD patients who are KT candidates that establishes the prevalence of frailty according to the FRAIL scale and factors associated. This sample represents about 50% of all patients over 50 years included in the KT waiting list in Spain (data provided by Spanish National Transplant Organization). Although less than 10% of KT candidates ≥50 years old in Spain are frail, the prevalence of pre-frailty and frailty together was almost 50% of the cohort. Female sex, comorbidity, and medications were strongly associated with frailty status.

The prevalence of frailty among KT candidates already listed for transplantation may vary from less than 5% to more than 50%, depending on the population and the scale used [6–9]. A large multicenter study from the US identified 18% of individuals as frail at the time of initial evaluation, while only 12% of individuals were identified as being frail among those who were ultimately listed for KT [6]. Among KT recipients, frailty prevalence before transplantation was established at 17.1% when a pooled analysis was made [10]. However, eleven of the fourteen studies included in the analysis were from the US. In Europe, two single-center studies have explored the prevalence of frailty in KT candidates/recipients: 15% of KT recipients were frail according to the Groningen Frailty Indicator in a Dutch study [11], and 10.5% and 3.6% of KT candidates were frail according to the PFP and the FRAIL scale, respectively, in a Spanish study [7]. In this multicenter study, we describe a large cohort of Spanish KT candidates over 50 years listed for transplantation with a prevalence of frailty of 8.2% according to the FRAIL scale. Pre-frailty was a very frequent finding, with 41.5% of candidates scoring 1 or 2 points by FRAIL. This has relevance as not only frailty but also pre-frailty has been associated with poorer outcomes in patients after transplantation [20]. In our cohort, the most frequently reported item was fatigue, followed by loss of weight. The lack of robustness was present in 15.5% of the patients. The latter is especially relevant given that pre-transplant grip strength has been found to be the most important frailty item related to post-transplant outcomes [34].

Differences in frailty prevalence may respond to different scales applied. Although there is an agreement regarding the underlying conceptual framework of frailty, there is a low level of consensus regarding the constituent elements to be included in operational definitions of frailty [35]. Consequently, various frailty metrics, encompassing different aspects like physical reserve, morbidity, cognition, or social factors, have been developed to date [3]. The PFP remains the most popular one for KT candidates and recipients and is characterized by the presence of three out of five indicators: slow walking speed, low physical activity, unintentional weight loss, weakness, and exhaustion. It has been suggested as the preferred choice for measuring physical reserve [36]. In contrast, the FRAIL scale also requires 3 out of 5 criteria—weight loss, resistance, fatigue, ambulation, and illness—but all items are self-reported [25], and, therefore, easier and faster to apply in the clinical practice setting. On the other hand, as the FRAIL scale did not account for objective measurements of physical reserve, it might underestimate the presence of frailty and classify as robust a patient who can be pre-frail or frail [28]. The decision regarding which scale to utilize during candidate evaluation will hinge on various factors, including the scale’s feasibility concerning time and resource consumption. In any case, clinicians should opt for a validated frailty scale, as they have demonstrated better transplant outcomes [26]. In our study, the prevalence of frailty was lower than the reported in studies from the US (8.2% vs. 15%–20%). This may reflect population differences, but also scale-dependent differences, as FRAIL usually estimates a lower prevalence of frailty than others that include physical domains [28]. There is no clear consensus on what frailty tool should be used in this population, and no systematic determinations are held during KT candidates’ evaluation [37]. Reasons to choose one frailty tool over the rest are broad, and KT candidates lack of a specific frailty tool (in contrast to liver transplant candidates) [38]. We chose the FRAIL scale because at that time 90% of KT centers in Spain were not systematically measuring frailty in their KT candidates. FRAIL scale has been acknowledged as a validated screening tool for frailty and is very easy to implement [39]. Our aim was to dimension and highlight the problem of frailty, and we needed to establish the frailty prevalence with a tool that most of the centers were willing and able to do.

Despite being a geriatric syndrome, age was not related to frailty in KT candidates, similar to what other studies in the CKD population have found [2, 40, 41]. Additionally, this fact could be related to a more restrictive selection of older candidates included in the waiting list [42]. We did find that female sex and comorbidity/treatment are associated with frailty in our cohort of patients. The second one is foreseeable as the FRAIL scale accounts for disease as part of its frailty phenotype. Importantly, cardiovascular disease seems to play the leading role in this association. On the contrary, time on dialysis was not associated with frailty, despite frail patients presented with a substantial longer time on dialysis. Although patient’s functional status decline seem irreversible after starting dialysis, studies have reported improvement in frailty status in up to one third of patients after starting dialysis [43]. Regarding sex, studies in community-dwelling populations have revealed a higher prevalence of frailty in females compared to males [44]. Studies including liver and kidney transplant candidates have found similar results [40, 45]. However, although women present with more frailty than men do, health results in the general population are usually worse in the latter, known as the male-female health-survival paradox [44, 46]. In liver transplant candidates, however, women present with higher mortality rates on the waiting list [45], while female kidney transplant candidates have lower mortality rates than men [47, 48]. Moreover, not only the prevalence but also the components and characteristics of frailty differ between male and female frail patients [40]. Examining sex-based disparities in frailty holds the potential to enhance risk assessment before transplantation and tailor specific, personalized interventions. Regarding BMI and albumin levels, we did not find any association with frailty status in this cohort. This may reflect how poorly BMI and albumin levels detect potential sarcopenia in these patients. Conversely, a higher BMI was found among frail patients. As sarcopenia is defined as reduced muscle mass and strength, a higher BMI does not necessarily reflect less risk of sarcopenia [49]. Moreover, it has been described that albumin levels may not differ among robust and frail KT candidates but sarcopenia does [28]. Novel biomarkers should guide the future investigation in this regard [50].

Our study has limitations, as it is a cross-sectional study that analyzes frailty prevalence and factors associated, lacking a follow-up of the patients. Regarding the study design, it has a potential selection bias, as there were centers with a high number of patients included while others only included a few patients. In addition, the FRAIL scale has been proposed as a screening frailty tool [23, 31], and its sensitivity detecting CKD frail patients may be lower [7, 28, 39]. However, this is to our knowledge the largest cohort of European KT candidates with frailty measurement reported so far. We aimed to establish the dimensions of the frailty problem in the KT waiting list in Spain, using a representative cohort. More than 1,000 patients over 50 years have been analyzed, from a total (considering all ages) of 4000 individuals included in the KT waiting list in Spain by the end of 2023 [22]. We provide important information about the prevalence and factors associated with frailty that may serve to implement adequate preventive and treatment interventions in this population. The photograph of the situation might be also useful for changing health policies.

In conclusion, less than 10% of the KT waiting list in Spain is frail according to the FRAIL scale, but pre-frailty and frailty together account for half of the patients. Female sex and comorbidity burden are factors associated with frailty. As frailty has a negative impact on outcomes after transplantation, measurements to improve/revert frailty should be part of the healthcare and preparation of candidates for KT.

## Data Availability

The raw data supporting the conclusions of this article will be made available by the authors, without undue reservation.
